# The Impact of Hepatitis C Virus Genotypes on Oxidative Stress Markers and Catalase Activity

**DOI:** 10.1155/2021/6676057

**Published:** 2021-02-25

**Authors:** Vukica Đorđević, Dobrila Stanković Đorđević, Branislava Kocić, Marina Dinić, Danka Sokolović, Jana Pešić Stanković

**Affiliations:** ^1^Faculty of Medicine University of Niš, Serbia; ^2^Clinical Centre of Niš, Serbia; ^3^Institute of Public Health Niš, Serbia

## Abstract

Hepatitis C virus (HCV) is a major cause of liver disease worldwide. Chronic HCV infections are usually associated with increased oxidative stress in the liver tissue. The intensity of oxidative stress may be a detrimental factor in liver injury and may determine the severity of the disease. The aim of the present case-control study was to determine the level of lipid peroxidation (TBARS), protein oxidative modification (AOPP), and catalase activity in sera of patients infected with HCV, in relation to different HCV genotypes and viral load. Considering the HCV patients with chronic hepatitis C (52) and control subject (50) recruitment, the study was designed as a case–control-type. The HCV RNA isolation, viral load, and HCV genotyping were performed according to the standard protocols. A significant difference compared to control healthy subjects was reported for TBAR (*p* < 0.001), AOPP (*p* = 0.001), and catalase activity (*p* = 0.007). In a gender-based comparison, a significantly higher level of AOPP for females was reported (*p* < 0.001). As stratified by HCV genotype, the most common was HCV-1 (HCV-1a and HCV 1b), with the overall participation of more than 60%, followed by genotype 3, while the least represented was genotype 2. No significant difference was documented among genotypes in regard to oxidative stress markers, although somewhat higher TBARS level, but not significant, was registered in patients infected with genotype 1b. A statistically significant positive correlation was found between the concentration of HCV genome copies and AOPP (*r* = 0.344; *p* = 0.012). A high level of HCV viral load was more likely to have a higher TBARS, but still without statistical significance (*p* = 0.072). In conclusion, the results obtained confirmed an imbalance between the ROS production and antioxidative defense system in HCV-infected patients. Since oxidative stress may have a profound influence on disease progression, fibrosis, and carcinogenesis, our results may meet the aspirations of mandatory introduction of antioxidants as early HCV therapy to counteract ROS consequences.

## 1. Introduction

Hepatitis C virus (HCV) is a major cause of liver disease worldwide. According to the World Health Organization (WHO) report, the estimated prevalence of 0.5%-3% is registered worldwide. In Europe, it is estimated that there are about 14 million people infected [1]. Hepatitis C virus (HCV) belongs to the *Flaviviridae* family, genus *Hepacivirus*. HCV is a small (55-65 nm in diameter), enveloped, icosahedral particle. The genome of the virus is positive single-stranded RNA with approximately 9600 nucleotides in length. It is composed of two conserved untranslated terminal regions at the 5′ and 3′ ends and the open reading frame (ORF) in the middle. ORF is divided into structural genes (C, E1, and E2) and nonstructural genes (NS2, NS3, NS4a, NS4b, NS5a, and NS5b). The C gene encodes nucleocapsid protein; E1 and E2 genes encode glycosylated proteins of the envelope membrane; nonstructural genes encode nonstructural proteins, which participate in the processes of virus replication [2]. According to the level of nucleotide sequence homology, HCV isolates are divided into seven genotypes. The isolates, which belong to the same genotype, are further divided into subtypes (a, b, c, *etc*.) [3]. The degree of homology of nucleotide sequences between different genotypes is about 65% [4]. The region 5′, the core and nonstructural genes are highly conserved sequences of the viral genome, while E1 and E2 genes show a high nucleotide sequence heterogeneity among isolates of different genotypes [4]. In addition, a very important biological characteristic of HCV is the variability of its E2 gene domains HVR1 and HVR2. Mutations in the E2 gene lead to the appearance of HCV mutants (quasispecies). Since the E2 protein is a target for neutralizing antibodies, mutations of this HCV region enable the virus to escape the elimination by the humoral immune response. This is explained as the mechanism of establishing a persistent infection [5].

The HCV infection is a serious health problem, because of a persistent infection in most infected individuals. It may be clinically manifested by chronic liver disease, often with a progressive course, from chronic hepatitis, through liver cirrhosis, to primary hepatocellular carcinoma. About 20 to 30% of patients with chronic hepatitis develop cirrhosis after 10 to 30 years. In these patients, the risk of death from cirrhosis-related complications is 4% per year, and the risk of developing a hepatocellular carcinoma (HCC) in cirrhotic patients is 1% to 5% per year. Thirty-three percent of patients with HCC die within one year after diagnosis [6, 7]. The pathogenetic mechanisms responsible for hepatocyte damage include cytotoxic T cell immune response and oxidative stress [8].

Chronic hepatitis C virus (HCV) infections are usually associated with increased oxidative stress in the liver tissue. The intensity of oxidative stress may be a detrimental factor in liver injury and may determine the severity of the disease [9, 10]. The cells responsible for ROS production and liberation are hepatocytes, nonparenchymal liver cells, such as Kupffer cells (resident macrophages), inflammatory cells, hepatic stellate cells (HSCs), and other immune effector cells [11]. They interact with each other, and a vicious cycle arises. Following liver injury, a set of reactive oxygen species (ROS) is released throughout the disturbance of mitochondrial electron transfer chain and oxidase activity [12]. Among the increased rate of ROS, species are the superoxide anions (O_2_^−^), hydrogen peroxide (H_2_O_2_), and the most hazardous hydroxyl radical (HO∙). It may lead to a serious disintegration of cell lipid components (phospholipid membranes), the oxidative modification of proteins, and oxidative modification of DNA. They may induce cell damage, protein functional deficiency, and genome instability, associated with the increased incidence of cell death and/or hepatocellular carcinoma. Hepatic fibrosis is a complex process, which involves the death of hepatocytes and activation of hepatic stellate cells [13].

Liver cells possess a specialized system of defense against oxidative stress, which comprises enzymes and nonenzymatic system of vitamin and nonvitamin origin. Among the enzymatic systems in the liver tissue, the most pronounced are catalase, superoxide dismutase (SOD), glutathione reductase (GSR), and glutathione transferase (GST). Consequent cell damage may lead to a short-term adaptation via induced liver regeneration, but very quickly, the adaptive mechanisms are reduced and the two possible pathways may arise, i.e., genomic instability, which may lead to carcinogenesis or to cell death. As a consequence of altered protein synthesis and consequent protein oxidative modification, the antioxidative defense enzymatic system becomes relatively insufficient to protect against the enormous attack of liberated ROS [14, 15]. Cell death induces the replacement of functional liver tissue by fibroblasts and in consequence functional liver failure and cirrhosis begin. ROS may play an important role as signal molecules responsible for the activation of stress-surviving signaling pathways and the redox-sensitive transcriptional factors. It may further induce inflammation and consequent free radical release from the next actor-inflammatory cells. Among them are Kupffer cells, which secrete interleukin- (IL-) 1𝛽, tumor necrosis factor-*α* (TNF-*α*), TGF-beta, and release ROS by activation of NADPH oxidase. ROS production induces the activation of redox-sensitive proinflammatory transcription factor NF-𝜅B. Released free radicals activate hepatic stellate cells further, which become the next player and important source of ROS responsible for further hepatocyte damage and induction of the fibrosis process [16, 17].

The aim of the present case-control study was to determine the level of lipid peroxidation (TBARS), protein oxidative modification (AOPP), and catalase activity in serum of patients infected with HCV, in relation to different HCV genotypes and viral load.

## 2. Patients and Methods

Considering the patients with chronic hepatitis C (52) and control subject (50) recruitment, the study was designed as a case–control-type. The study was approved by the Ethical Committee of Faculty of Medicine (Decision no. 12-6972-2/6). All subjects recruited for the study signed the informed consent. The investigation was performed at the Institute for Public Health and Faculty of Medicine University of Niš (Serbia).

The enrolled patients, besides being HCV positive, were recruited by specific eligibility criteria, including the absence of HBV infection, HIV infection, other chronic liver diseases, any acute or chronic systemic immunologic conditions and inflammatory diseases, cancer, cardiovascular, or kidney diseases. The control group comprised 50 healthy volunteers, who met the same eligibility criteria, i.e., they were HCV negative, and were age- and gender-matched.

The diagnosis of HCV was performed according to the standard diagnostic protocols. The HCV RNA isolation was performed by kit Ribo-virus Sacace. Plasma HCV RNA was detected using HCV REAL-TM QUAL new version Sacace. For the detection of HCV RNA concentration (IU/mL of plasma)-viral load, HCV REAL-TM QUANT new version Sacace was used. HCV genotyping was performed using HCV Genotype PLUS REAL-TN, Sacace. All the diagnostic procedures were performed according to the recommendations of the manufacturer.

For the analysis, morning blood samples from HCV patients and control subjects were taken and centrifuged at 3.000 rpm; serum samples were kept at −80°C.

The concentration of TBARS-reactive compounds (MDA level) was performed according to the method described by Sahreen et al. [18], based on the determination of malondialdehyde (MDA), which reacts with the thiobarbituric acid, forming a pink complex. The reaction mixture contained 0.2 mL of serum, 0.2 mL of the ascorbic acid (100 mM), 0.58 mL of the potassium phosphate buffer (0.1 M; pH = 7.4), 0.02 mL of the Ferric chloride-FeCl3 (100 mM), 1 mL of the CCl3COOH (10%), and 1 mL of the TBA (0.67% dissolved in 0.1 M NaOH) and heated for 30 min in a boiling water bath (100°C). After the centrifugation at 4000 rpm for 10 min, the absorbance of pink color was read at 535 nm. The concentration of the TBARS was expressed in *μ*mol/L MDA.

The concentration of AOPP was determined by spectrophotometric method according to the method of Witko-Sarsat et al. [19]. The quantity of 200 *μ*L of serum, diluted as 1 : 5 in PBS, and chloramine-T standard solutions were placed in a 96-well microtiter plate, followed by 20 *μ*L of acetic acid. Ten microliters of 1.16 M KJ was added, followed by 20 *μ*L of the glacial CH3COOH. The yellow color was read at 340 nm in a microplate reader against a blank. The concentration of the AOPP was expressed in *μ*mol/L chloramine T.

Catalase activity in serum was determined using a spectrophotometric method of Nabavi et al. [20]. Briefly, a reaction mixture contained 0.5 mL of 50 mM phosphate buffer (pH 5.0), 1.5 mL of 5.9 mM H_2_O_2_, and 0.1 mL of serum. It was incubated for 5 min; afterward, the reaction was stopped by adding 0.1 mL of 20% TCA and 1 mL of 4% ammonium molybdate. The samples were centrifuged and, the change in absorbance was calculated at 240 nm, by subtracting from the standard sample, where 0.1 mL of distilled water was added instead of serum. CAT activity was defined as Kat/L, meaning an absorbance change of 0.01 as unit/min.

Statistical data analysis was performed in the SPSS program, version 20. The normality of data distribution was tested by the Kolmogorov-Smirnov test. The comparison between groups was performed by the Student's *t*-test and ANOVA in the case of normally distributed data. In the case when the data distribution was not normal, Mann–Whitney *U* test or Kruskal-Wallis test was used. The correlation between the variables was performed by the Pearson coefficient of the linear correlation. Statistical significance is considered for *p* < 0.05.

## 3. Results

Table 1 shows the HCV genotype distribution among patients. As stratified by HCV genotype, the distribution of the study population was as follows: the most common was HCV-1 (HCV-1a and HCV 1b), with the overall participation of HCV-1 more than 60%, followed by genotype 3, while the least represented was genotype 2.

Table 2 shows the level of oxidative stress parameters (TBARS, AOPP) and catalase activity in HCV patients and control healthy subjects. All parameters examined showed a significant difference compared to control healthy subjects, which for TBAR was as *p* < 0.001, for AOPP as *p* = 0.001, and for catalase as *p* = 0.007.

Table 3 shows a gender-based comparison of oxidative stress parameters (TBARS and AOPP) and catalase activity in the HCV patient group. A significantly higher level of AOPP for females was reported, which was as *p* < 0.001. Additionally, a gender distribution showed that males were more likely to have a higher TBARS level and catalase activity, but without any statistical significance.

The values of oxidative stress parameters (TBARS and AOPP) and catalase activity in patients infected with different HCV genotypes are summarized in Table 4. Genotype distribution revealed that HCV1b patients were more likely to have a higher TBARS compared to others, HCV 3 patients were more likely to have a higher AOPP level, while patients infected with HCV1a were more likely to have a low catalase activity, but without statistical significance.

Table 5 shows the correlation of HCV viral load and oxidative stress parameters (TBARS and AOPP) and catalase activity. A statistically significant positive correlation was found between virus genome copies concentration and AOPP (*r* = 0.344; *p* = 0.012), which means that with the increase in the concentration of gene copies the level of AOPP increases significantly. Correlation analysis showed that the high level of HCV viral load was more likely to have a higher TBARS, but without statistical significance (*p* = 0.072).

## 4. Discussion

Our study documented that among all HCV genotypes detected, genotype 1 (a and b) represents the most prevalent genotype with the distribution of over 60%, followed by genotype 3 (Table 1). This frequency distribution is reported worldwide [4].

The present study further involved the evaluation of oxidative stress markers (TBARS and AOPP) and catalase activity in patients infected with HCV genotypes 1a, 1b, 2, and 3. The results obtained indicated that patients infected with different genotypes were under altered equilibrium between the ROS production and antioxidant defense system, due to overproduction of ROS and a decreased antioxidative defense. The level of TBAR-reacting substances, expressed as MDA level, was almost twice as that of the uninfected, healthy controls (Table 2). A gender-based comparison of oxidative stress parameters (TBARS and AOPP) and catalase activity in the HCV patients group revealed a significantly higher level of AOPP for females, while males were more likely to have a higher TBARS level, but without statistical significance (Table 3).

No significant difference was documented among genotypes, although somewhat higher TBARS level, but not significant, was registered in patients infected with genotype 1b (Table 4). Our results are in accordance with Ansari et al. [21] and Limongi et al. [22], in which only subtle changes in MDA level in groups of 1a and 1b genotype have been documented [21].

The HCV genotype studies have been mainly oriented toward the evaluation of their correlation with the severity of the disease, the appearance of fibrosis, carcinogenesis, and resistance to therapy. The increase in ROS production and their consequences on lipid peroxidation and protein oxidative modification are among the mechanisms which have been implicated in the end-stage cirrhosis following the HCV infection [23–25]. As our study suggests, the correlation of viral load and oxidative stress parameter AOPP revealed a statistically significant positive correlation (Table 5). This result suggests that viral load can be directly responsible for ROS production and liberation and consequent cell damage. Since the correlation analysis showed that the high level of HCV viral load was more likely to have a higher TBARS, it may suggest that viral load can also have an impact on membrane damage and lipid peroxidation.

It is possible that HCV genome-encoded nonstructural proteins have a role in pathogenetic mechanisms responsible for excessive ROS release in hepatocytes. Among them, the best explained is a NS5A, which is a multifunctional protein and it contributes to the HCV replication. Besides the central role of NS5A in HCV replication, it is capable of inducing IFN resistance, through the repression of PKR function. The mechanisms of triggering the liberation of ROS by NS5A are complex, finally leading to consequent activation of inflammatory, redox-sensitive NF-*κ* B, and AP-1 transcription factors. Proinflammatory transcription factor NF-*κ*B has been declared the central mediator of cell immune and inflammatory response. Its active subunits, comprising the NF-*κ*B1 (p50, p105), NF-*κ*B2 (p52, p100), RelA (p65), RelB, and c-Rel, induce the expression of many genes, with consequent synthesis of a number of inflammatory cytokines, such as tumor necrosis factor-*α* (TNF-*α*), IL-1, IL-6, IL-18, lymphotoxin CXCL4, IFN-*γ*, chemokines, and TGF-*β* [26, 27]. It is very interesting to note that generated ROS although HCV NS protein reaction products, in turn, may suppress HCV replication [23]. A recent publication documented that HCV core protein may induce inflammasome activation and IL-1*β* release from hepatic macrophages [28]. The precise mechanisms for this have been documented by using an *in vitro* model of Huh-7 cell culture, transfected with NS5A, where untransfected tissue served as control. By studying the existing NS5A three domains involvement in ROS production, it was reported that besides full-length NS5A protein, its domain I may exclusively induce ROS production [29]. As a possible mechanism of NS5A-induced release of ROS is a direct NS5A-induced damage of intracellular membrane components, including ER, peroxisomes, and mitochondria. After the association with membrane components, it induces ER stress, followed by a simultaneous efflux of Ca^2+^ ions from the ER. They can be taken up by mitochondria, altering in that way a transmembrane potential, followed by a reduction of molecular oxygen, which induces the accumulation of unstable superoxide anion radical (O^2−^) in mitochondria. In this pathway, the mitochondrial oxidative phosphorylation chain located on the inner mitochondrial membrane is the main source of free radical generation [30–32].

Besides the abovementioned mitochondrial mechanism of NS5A-induced release of ROS, the expression of NADPH oxidase family (NOX1 and NOX4) and cyclooxygenase 2 (COX-2) have been shown to occur simultaneously. As in the case of the mitochondrial pathway, it was reported that the domain I can induce NOX1. Specific cascade of their effect lead to the conclusion that all systems can act in accord with a specific order, which includes TGF*β*1, NOX1, COX-2, and NOX4. In that context, the next player involved in active ROS generation seems to be a Cytochrome P450 2E1 (CYP2E1) which has a typical localisation in the ER membrane. Besides NS5A, the effect on NOX4 may exert HCV structural proteins as well [29]. The importance of NADPH oxidase and CYP2E1 was documented in the development of chronic inflammation and hepatocyte death. Since the activation of fibroblasts migration and excessive collagen deposition overcome liver regenerative capacity, consequent liver fibrosis seems to be a necessary harm [29, 33, 34].

Released ROS may damage susceptible cell biomolecules and related cell structures, such as unsaturated free fatty acids of membrane phospholipids, proteins of structural and functional cellular components, such as enzymes and receptors, and DNA. As concerns the cell membranes, generated ROS can alter the structure and function of cell organelles and outer cell membrane, leading to peroxidative breakdown of membrane phospholipids. The release of lysosomal hydrolytic enzymes can further aggravate liver cell damage. The released damaged particles may further act as the DAMP molecules (damage-associated molecular patterns), activating HSCs, which proliferate and belong to the transformation into myofibroblast-like cells. Following the transformation, they exert fibrogenic potential, by secreting collagen type I, III, and IV, fibronectin, laminin, proteoglycans, and transforming growth factor-*β* (TGF-*β*). In this way, they fill irreversibly the empty spaces of dead hepatocytes. Inflammatory cytokines, released from the surrounding inflammatory cells, may further produce ROS [35, 36]. ROS has the potential to decrease tumor-suppressor p53 protein expression [14, 15], stimulating a possible carcinogenesis. The values of oxidative stress parameters (TBARS and AOPP) and catalase activity in diverse to HCV genotypes (Table 4) may suggest that among the genotype distribution, the HCV1b patients were more likely to have a higher TBARS compared to others, HCV 3 patients were more likely to have a higher AOPP level, while patients infected with HCV1a were more likely to have a low catalase activity, but without statistical significance. The absence of a statistically significant difference in the production of ROS between the genotypes can be explained by the fact that nonstructural viral proteins have a high level of homogeneity between different genotypes.

Considering the activity of catalase in relation to the single genotypes, a trend of significantly decreased catalase activity was observed in each of the presented genotypes. Liver cells develop different protective mechanisms to prevent ROS effects. It is well-documented that among the antioxidative enzymes, the activity of catalase is very high in liver tissue. In this way, it provides the first line of antioxidative defense enzymatic system. In the case of chronic hepatitis C, a number of results documented decreased antioxidative defense system, expressed as total antioxidative capacity or the low level of reduced glutathione. Decreased antioxidative capacity in view of catalase activity brings about the inability of liver tissues to counteract oxidative stress or persistent and chronic tissue damage [21, 23].

In this way, both increased ROS production and decreased antioxidative defense are working in accord with inflammation to produce cell damage and hepatic genome instability. Our results are consistent with the results of other studies, concerning the level of nonenzymatic antioxidative defense level (GSH) [33]. The activity of antioxidative enzymes may vary, depending on preserved compensatory mechanisms. Some reports discussed the increased activity of antioxidant enzymes MnSOD and catalase in HCV infections [37–39].

In conclusion, the results obtained confirmed an imbalance between the ROS production and antioxidative defense system in HCV infected patients, with a markedly enhanced lipid peroxidation and protein oxidative modification, followed by the reduction of catalase activity. Since oxidative stress may have a profound influence on disease progression, fibrosis, and carcinogenesis, our results may meet the aspirations of mandatory introduction of antioxidants as early HCV therapy to counteract ROS consequences.

## Figures and Tables

**Table 1:** Genotype distribution among HCV patients.

	*n*	%
1a	17	33.3
1b	14	27.5
2	4	7.8
3	16	31.4

HCV genotyping was performed using HCV Genotype PLUS REAL-TN.

**Table 2:** Oxidative stress parameters (TBARS and AOPP) and catalase activity between HCV patients and control healthy group.

	HCV patients	Control group	*t*/*Z*	*p*
TBARS	6.99 ± 4.02	4.38 ± 1.38	3.712	<0.001
AOPP	122.40 ± 43.12	97.92 ± 30.90	3.285	0.001
Catalase	183.41 ± 134.08	260.60 ± 132.04	2.677	0.007

Data are expressed as mean ± SD.TBARS were expressed as (*μ*mol/LMDA), AOPP as *μ*mol/L chloramine T, and catalase as (Kat/L). Statistical analysis was performed by using *Z*- Mann–Whitney *U* test.

**Table 3:** Gender-based comparison of oxidative stress parameters (TBARS and AOPP) and catalase activity in the HCV patient group.

	Man	Women	*t*/*Z*	*p*
TBARS	7.72 ± 5.00	6.55 ± 3.26	3.712	0.522
AOPP	107.42 ± 40.38	131.74 ± 42.72	2.034	0.047
Catalase	195.84 ± 118.33	174.67 ± 145.69	2.677	0.409

Data are expressed as mean ± SD. TBARS were expressed as (*μ*mol/LMDA), AOPP as *μ*mol/L chloramine T, and catalase as (Kat/L). Statistical analysis was performed by using *Z*- Mann–Whitney *U* test.

**Table 4:** Oxidative stress parameters (TBARS and AOPP) and catalase activity in regard to HCV genotype.

	TBARS	AOPP	Catalase
1a	6.73 ± 3.11	117.27 ± 33.84	172.17 ± 149.34
1b	7.45 ± 5.86	122.46 ± 48.53	190.92 ± 137.66
2	6.93 ± 4.47	124.14 ± 43.41	192.00 ± 180.68
3	6.73 ± 3.15	125.87 ± 50.98	182.38 ± 125.25
F/*χ*_KW_^2^	0.180	0.107	0.420
p	0.981	0.955	0.936

Data are expressed as mean ± SD. TBARS were expressed as (*μ*mol/LMDA), AOPP as *μ*mol/L chloramine T, and catalase as (Kat/L). Statistical analysis was performed by using F-ANOVA and *χ*_KW_^2^ –Kruskal-Wallis test.

**Table 5:** Correlations between HCV viral load and oxidative stress parameters.

		Viral load	TBARS	AOPP	Catalase
Viral load	*r*		0.251	0.344	0.020
	*p*	1	0.072	0.012	0.897
TBARS	*r*			0.098	0.189
	*p*		1	0.489	0.209
AOPP	*r*				0.101
	*p*			1	0504
Catalase	*r*				
	*p*				1

HCV RNA concentration (IU/mL of plasma)-viral load was correlated with TBARS, AOPP, and catalase by using *r*-Pearson correlation coefficient.

## Data Availability

The data that support the findings of this study are available from the corresponding author, [VD], upon request.
